# A Peculiar Form of Motor Disturbance of the Pupil

**Published:** 1886-12

**Authors:** J. Salgó

**Affiliations:** Central-blatt für Nerven Heilkunde


A PECULIAR FORM OF MOTOR DISTURBANCE OF THE PUPIL (*Eine Besondere Form von Bewegungsstörung der Pupille*). *By* DR. J. SALGÓ, *Central-blatt für Nerven Heilkunde. October, 1886.*

Salgó describes a peculiar form of pupil, which consists essentially of an irregular contraction of the muscular tissue of the iris, by which its pupillary margin assumes many different shapes. The pupil generally appears triangular or poliangular, with the corners thickened and rounded out, resembling somewhat the slit-like pupil of the cat, or the irregular appearance caused by synechia. This kind of pupil reacts in a normal manner, but after each movement assumes a somewhat different form, so that the contracted pupil appears different from the same when dilated.

The great majority of cases so far observed have been associated with general paralysis of the insane, though it has been seen in chronic, progressive psychoses in which no paralysis could be detected. The author considers that it is much the most frequent pupillary symptom in general paralysis, and regards it as an expression of the varying intensity of enervation from the cortex. HAROLD N. MOYER.

